# Characterization of the complete chloroplast genome of *Lonicera japonica* (Caprifoliaceae), a long history herb species plant from China

**DOI:** 10.1080/23802359.2019.1687353

**Published:** 2019-11-12

**Authors:** Jiang Luo

**Affiliations:** Jiangxi Province Hospital of Integrated Chinese and Western Medicine, Nanchang, P. R. China

**Keywords:** *Lonicera japonica*, Caprifoliaceae, chloroplast genome, phylogenetic relationship

## Abstract

*Lonicera japonica* is a common ornamental and medicinal plant in North America and East Asia. In this study, the complete chloroplast genome of *L. japonica* was presented and annotated. The chloroplast genome of *L. japonica* is 155,078 bp in length, which has a large single-copy (LSC) region of 88,859 bp, a small single-copy region (SSC) of 18,647 bp, and a pair of inverted-repeat (IRs) regions of 23,786 bp in each one. The overall nucleotide composition is 30.2% of A, 31.2% of T, 19.6% C, and 19.0% G, with a total A + T content of the chloroplast genome 61.4% and G + C content of 38.6%. The chloroplast genome of *L. japonica* contains 130 genes, including 83 protein-coding genes (PCGs), 39 transfer RNA (tRNAs), and 8 ribosome RNA (rRNAs). Phylogenetic relationship used the maximum-likelihood (ML) method that *L. japonica* is closely related to *Lonicera macranthoides.* This study can use for medicinal valuable and clinical drug development for the future.

*Lonicera japonica* belongs to the Caprifoliaceae family and is often used in traditional Chinese and Japanese medicine (Shang et al. 2011). It is widely cultivated in North America and East Asia including China, Japan, and Korea as an effective groundcover because of its pleasant, sweet-smelling flowers (Xia et al. 2016). In China, the *L. japonica* (Chinese name ‘Jin-Yin-Hua’) and has been an important medicinal plant for thousands of years, which was cultivated as medicinal plant with great economic value in the pharmaceutical industry (Muluye et al. 2014). Modern pharmacological studies have shown that extracts from *L. japonica* possess a wide range of bioactive properties, such as anti-bacterial, anti-inflammatory, antiviral, anti-pyretic, anti-oxidant, anti-hyperlipidemic, and anti-nociceptive among others (Li et al. 2015). In order to further study, the phylogenetic relationship of *L. japonica*, we presented the complete chloroplast genome of *L. japonica*, which can use for medicinal valuable and clinical drug development in further.

The specimen sample of *L. japonica* was collected from Jiangxi Province Hospital of Integrated Chinese and Western Medicine (Nanchang, Jiangxi, China, 115.91E; 28.67N). Total genomic DNA of *L. japonica* was extracted from the fresh flowers using Plant Tissues Genomic DNA Extraction Kit (Solarbio, Beijing, China) and stored in Jiangxi Province Hospital of Integrated Chinese and Western Medicine (No. JXPHICWM03). The chloroplast (cp) DNA was purified and fragmented using the NEB Next Ultra^TM^ II DNA Library Prep Kit (NEB, Beijing, China), which was sequenced. Quality control was performed to remove low-quality reads and adapters using the FastQC software (Andrews 2015). The chloroplast genome was assembled and annotated using the MitoZ software (Meng et al. 2019). The physical map of the chloroplast genome was generated using OrganellarGenomeDRAW (Lohse et al. 2013).

The complete chloroplast genome of *L. japonica* (GenBank with accession No. MK9677892) is with 155,078 base pairs (bp) in length as the circular, which has a characteristic quadripartite structure with a large single-copy region (LSC) of 88,859 bp, a small single-copy region (SSC) of 18,647 bp, and a pair of inverted-repeat regions (IRs) of 23,786 bp. The chloroplast genome of *L. japonica* contains 130 genes, including 83 protein-coding genes (PCGs), 39 transfer RNA genes (tRNAs), and 8 ribosomal RNA genes (rRNAs). Sixteen genes were found duplicated in each IR region, which was including four PCG species (*ycf2, ndhB, rps7,* and *rps12*), eight tRNA species (*trnH-GUG, trnL-CAA, trnV-GAC, trnI-GAU, trnA-UGC, trnR-ACG, trnG-UCC,* and *trnN-GUU*), and four rRNA species (*rRNA16, rRNA23, rRNA4.5,* and *rRNA5*). The overall nucleotide composition is 30.2% of A, 31.2% of T, 19.6% of C, and 19.0% of G, with a total A + T content of 61.4% and G + C content of 38.6%.

In this study, the maximum-likelihood (ML) method was used to analysis phylogenetic relationship of 11 the family Caprifoliaceae species plants with *L. japonica*. The phylogenetic tree was reconstructed using the MEGA X software with 2000 bootstrap values replicate at each node based on GTR model. All of the nodes were inferred with strong support by the ML methods. The final tree was represented using the MEGA X software (Kumar et al. 2018) and edited using the iTOL version 4.0 online web (https://itol.embl.de/) (Letunic and Bork 2016). Phylogenetic relationship (Figure 1) result has shown that *L. japonica* is closely related to *Lonicera macranthoides* (GenBank No. MH579750.1). This study is very important for the conservation and evolutionary and also can use for medicinal valuable and clinical drug development for the future.

**Figure 1.**
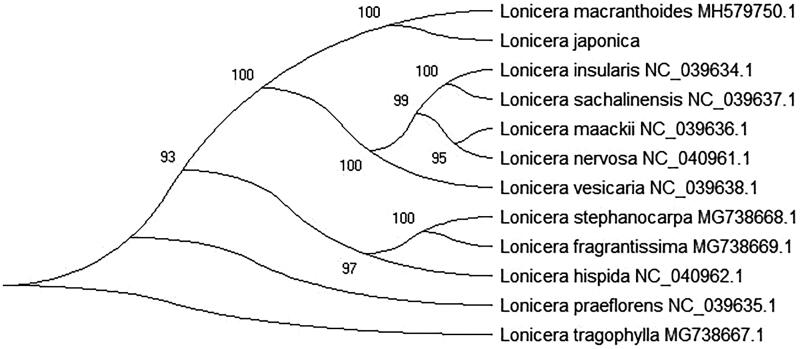
The maximum-likelihood (ML) phylogenetic tree was constructed using 12 family Caprifoliaceae plants chloroplast genomes data. The GenBank accession numbers are in the figure.

## References

[CIT0001] Andrews S 2015 FastQC: a quality control tool for high throughput sequence data. http://www.bioinformatics.babraham.ac.uk/projects/fastqc/.

[CIT0002] Kumar S, Stecher G, Li M, Knyaz C, Tamura K 2018 MEGA X: molecular evolutionary genetics analysis across computing platforms. Mol Biol Evol. 35(6):1547–1549. 29722887 10.1093/molbev/msy096 PMC5967553

[CIT0003] Letunic I, Bork P 2016 Interactive tree of life (iTOL) v3: an online tool for the display and annotation of phylogenetic and other trees. Nucleic Acids Res. 44(W1):W242–W245. 27095192 10.1093/nar/gkw290 PMC4987883

[CIT0004] Li YJ, Cai WY, Weng XG, Li Q, Wang YJ, Chen Y, Zhang W, Yang Q, Guo Y, Zhu XX, et al. 2015 *Lonicerae japonicae* flos and lonicerae flos: a systematic pharmacology review. Evid Based Complement Alternat Med. 2015:1–16. 10.1155/2015/905063 PMC4519546 26257818

[CIT0005] Lohse M, Drechsel O, Kahlau S, Bock R 2013 OrganellarGenomeDRAW–a suite of tools for generating physical maps of plastid and mitochondrial genomes and visualizing expression data sets. Nucleic Acids Res. 41(W1):W575–W581. 23609545 10.1093/nar/gkt289 PMC3692101

[CIT0006] Meng GL, Li YY, Yang CT, Liu SL 2019 MitoZ: a toolkit for animal mitochondrial genome assembly, annotation, and visualization. Nucleic Acids Res. 47(11):e63. 30864657 10.1093/nar/gkz173 PMC6582343

[CIT0007] Muluye RA, Bian YH, Alemu PN 2014 Anti-inflammatory and antimicrobial effects of heat-clearing Chinese herbs: a current review. J Tradit Complement Med. 4(2):93–98. 24860732 10.4103/2225-4110.126635 PMC4003708

[CIT0008] Shang X, Pan H, Li M, Miao X, Ding H 2011 *Lonicera japonica* Thunb.: ethnopharmacology, phytochemistry and pharmacology of an important traditional Chinese medicine. J Ethnopharmacol. 138(1):1–21. 21864666 10.1016/j.jep.2011.08.016 PMC7127058

[CIT0009] Xia H, Zhang LB, Wu G, Fu CH, Long Y, Xiang J, Gan JP, Zhou YH, Yu LJ, Li MT 2016 Genome-wide identification and characterization of micro-RNAs and target genes in *Lonicera japonica*. PLoS One. 11(10):e0164140. 27711182 10.1371/journal.pone.0164140 PMC5053492

